# Evaluation of the Microbiological Performance and Potential Clinical Impact of New Rapid Molecular Assays for the Diagnosis of Bloodstream Infections

**DOI:** 10.3390/microorganisms13030616

**Published:** 2025-03-07

**Authors:** Mateo Tićac, Tanja Grubić Kezele, Maja Abram, Marina Bubonja-Šonje

**Affiliations:** 1 Department of Anestesiology and Intensive Care, Clinical Hospital Center Rijeka, 51000 Rijeka, Croatia; mateo.ticac@uniri.hr; 2 Department of Anesthesiology, Reanimatology, Intensive Care and Emergency Medicine, Faculty of Medicine, University of Rijeka, 51000 Rijeka, Croatia; 3 Department of Clinical Microbiology, Clinical Hospital Center Rijeka, 51000 Rijeka, Croatia; maja.abram@uniri.hr (M.A.); marina.bubonja@uniri.hr (M.B.-Š.); 4 Department of Physiology, Immunology and Pathophysiology, Faculty of Medicine, University of Rijeka, 51000 Rijeka, Croatia; 5 Department of Microbiology and Parasitology, Faculty of Medicine, University of Rijeka, 51000 Rijeka, Croatia

**Keywords:** antimicrobial resistance, blood culture, bloodstream infection, Molecular Mouse

## Abstract

Bloodstream infection (BSI) is a critical medical emergency associated with a high mortality rate. Rapid and accurate identification of the causative pathogen and the results of antimicrobial susceptibility testing are crucial for initiating appropriate antimicrobial therapy. The aim of this study was to evaluate the performance of a new rapid PCR Molecular Mouse System (MMS) for the identification of Gram-negative bacteria (GNB) and GNB resistance genes directly from a positive blood culture (BC). The validation of these rapid multiplex assays was carried out in a real hospital setting. A total of 80 BSI episodes were included in our study and the results were compared with culture-based methods. BC samples in which GNB had previously been detected microscopically and which originated from different hospital wards were analysed. The MMS GNB identification assay achieved a sensitivity of 98.7% and a specificity of 100% for the covered pathogens. In one BC sample, *Klebsiella aerogenes* was identified at the family level (*Enterobacteriaceae*) with MMS. However, in three polymicrobial samples, MMS identified bacteria that were not detected by culture-based methods (*Klebsiella pneumoniae*, *K. aerogenes* and *Stenotrophomonas maltophilia*). MMS also showed excellent overall performance in the detection of GNB resistance markers (100% sensitivity and 100% specificity). The type of extended-spectrum beta-lactamase (ESBL) resistance gene identified correctly with MMS was CTX-M-1/9 (n = 17/20), alone or in combination with SHV-type β-lactamase or with the different types of carbapenemase genes. MMS detected one carbapenemase gene of each type (KPC, NDM and OXA-23) and six OXA-48 genes. In addition, the colistin resistance gene *mcr-1* was detected in one positive BC with *Escherichia coli* (*E. coli*). The time to result was significantly shorter for MMS than for routine culture methods. A retrospective analysis of the patients’ medical records revealed that a change in empirical antimicrobial therapy would have been made in around half of the patients following the MMS results. These results support the use of MMS as a valuable complement to conventional culture methods for more rapid BSI diagnosis and adjustment of empirical therapy.

## 1. Introduction

Bloodstream infection (BSI) and sepsis remain a leading cause of death, requiring rapid and accurate diagnosis and treatment [1]. Blood culture (BC), followed by phenotypic identification and antimicrobial susceptibility testing (AST) using the broth microdilution method or disc diffusion, is the gold standard for determining the cause of BSI. The conventional diagnostic approach often has a turnaround time of one to several days, and every day of delay in initiating effective antibiotic therapy can lead to worse patient outcomes. For this reason, the majority of septic patients receive initial empirical antimicrobial therapy before the identification of the infecting microorganism and its antibiotic susceptibility. A common approach is to use broad-spectrum antibiotics with the aim of covering several possible pathogens. However, broad-spectrum antibiotic treatment may be associated with a higher risk of adverse events. Rhee et al. reported a higher mortality rate associated with unnecessarily broad empirical treatment in a cohort of patients with community-onset sepsis [2]. On the other hand, there is also a risk of ineffective empirical therapy due to the increasingly rapid spread of multidrug-resistant (MDR) bacteria [2,3,4].

The choice of appropriate empirical therapy for the treatment of BSI depends, among other factors, on local epidemiology. Antimicrobial resistance poses a significant public health challenge in Croatia, with notable impacts on morbidity and mortality. The rate of invasive extended-spectrum beta-lactamase (ESBL)-producing *Escherichia coli* and *Klebsiella pneumoniae* isolates is steadily increasing, reaching 27% and 54%, respectively, in Croatia in 2023 [5]. In addition, carbapenem resistance in invasive *K. pneumoniae* isolates reached 27%, with most strains producing the oxacillinase OXA-48-like type [6,7]. Carbapenem resistance is often combined with resistance to several other important antimicrobial classes, resulting in a severely limited range of treatment options. Of particular concern in recent years is the increase in carbapenem resistance in *Acinetobacter baumannii*, a typical hospital pathogen, which reached a high level of 97% [6]. We recently reported that the main reason for the inadequacy of empirical therapy in the cohort of septic patients hospitalised between 2020 and 2023 was the lack of specific coverage for ESBL-producing *Enterobacterales* and carbapenem-resistant Gram-negative bacteria (GNB) [8]. Faced with a high rate of antimicrobial resistance, clinicians now more frequently use empirical treatment with a very broad spectrum of activity and new commercially available antibiotics, accepting that, in many cases, this is unnecessary and may contribute to further resistance development.

In view of this, a more rapid microbiological diagnosis is required to expedite pathogen identification and the detection of resistance genes, thereby enabling earlier escalation of therapy for resistant organisms or the de-escalation of unnecessary broad-spectrum antibiotics. In recent years, several platforms have been marketed that enable molecular-based identification of bacterial and fungal pathogens directly from BC [9,10]. Previous studies have shown that the addition of rapid molecular biology methods to conventional microbiological methods and antimicrobial stewardship (AMS) practises in the diagnostic regimen of BSIs significantly reduces the time to optimal therapy [11,12]. Faster and more accurate identification of pathogens and their resistance profiles leads to more targeted and effective treatment regimens, helping to reduce unnecessary antibiotic use, curb antimicrobial resistance and ultimately improve patient care.

The performance of rapid, commercially available molecular technologies in BSI diagnostics is generally good when compared to conventional methods, except for polymicrobial samples [10]. However, the high cost of commercial assays limits their use in resource-limited settings.

The new CE IVD-labelled multiplex polymerase chain reaction (PCR) method, the Molecular Mouse System (MMS) (Alifax, Polverara, Italy), enables the rapid detection of BSI. Depending on the Gram-stain results, MMS offers the option to choose between different cartridges for the detection of a broad coverage of organisms. Starting from a positive BC and selecting a specific BC identification panel (Gram-positive bacteria, Gram-negative bacteria, GNB resistance markers, or yeast), results are provided in about 1 h [13]. Given the significantly higher local prevalence of MDR GNB compared to MDR Gram-positive bacteria, we aimed to evaluate a molecular-based sepsis panel for the detection of GNB and their resistance genes from positive BC.

In this study, we compared MMS assays with conventional culture-based methods in terms of (i) correct detection and identification of GNB, (ii) correct prediction of resistance genes, and (iii) potential impact of MMS results on the adjustment of empirical therapy.

## 2. Materials and Methods

### 2.1. Study Setting, Selection Criteria and Definitions

This prospective study was conducted from December 2024 to February 2025 at a Western Croatian clinical hospital centre with 1069 beds. BC samples were collected from patients hospitalised in different hospital wards (intensive care unit, infectious diseases ward, emergency department, paediatrics, general medicine wards, surgery). Only samples from adult patients (≥18 years old) were included in the study. The first bottle of each positive BSI episode was used. A total of 80 positively flagged BC bottles were included in the study. Only BC samples with Gram-negative bacteria were selected and analysed using both MMS and conventional microbiological methods. Conventional culture and MMS were performed in parallel, and the MMS result was not communicated to the prescribing physician.

A total of 20 BC samples with MDR GNB, confirmed by culture-based and molecular-based reference methods, were further selected for resistance markers analysis using the “MM gram neg res” assay. We selected BC samples with MDR GNB that are critical priority pathogens according to the Bacterial Priority Pathogens List of the World Health Organisation (WHO) [3].

MDR was defined as non-susceptibility to at least one agent from three or more antimicrobial categories. Empirical antibiotic therapy was considered adequate if the spectrum and dose were appropriate according to the organism and/or resistance gene detected.

All data used in study were fully anonymized. The study was conducted in the context of routine diagnostics using residual blood culture aliquots.

### 2.2. Diagnosis of BSI and Microbiological Tests

Incubation of BC bottles was performed in the automated Bact/Alert Virtuo BC system (bioMérieux, Marcy l’Etoile, France). Positive BactAlert FA/FN Plus BC bottles (bioMérieux, Marcy l’Etoile, France) are processed as soon as they are flagged positive according to the laboratory’s standard operating procedures. Direct identification of bacterial pellets from positive BC was carried out with the Vitek 2 system (bioMeriéux, Marcy l’Etoile, France) using ID-GN cards as described previously [14]. Thereafter, each positive bottle was subcultured onto solid agar media comprising 5% sheep blood (BD Difco, Le Pont-de-Claix, France) and incubated at 35 °C with 5% CO_2_ for 18–24 h. Identification of isolated colonies to species level was confirmed by Vitek 2 system (ID-GN cards) according to the manufacturer’s instructions. Identification was supplemented, when necessary, with FilmArray blood culture identification (BCID) panel on BioFire FilmArray Multiplex PCR instrument (bioMe’rieux, Marcy l’Etoile, France).

### 2.3. Antimicrobial Susceptibility Testing (AST)

Rapid antimicrobial susceptibility testing (RAST) directly from positive blood culture bottles based on the European Committee on Antimicrobial Susceptibility Testing (EUCAST) standard disc diffusion method was performed as proposed by EUCAST [15]. Inoculated Mueller–Hinton agar (bioMérieux) with applied antibiotic discs (Liofilchem, Roseto degli Abruzzi, Italy) were incubated 4 h. If needed, the plates were re-incubated to enable reading at a later time (6 h and/or 8 h). When it was not possible to read results after 4–8 h incubation due to limited opening hours of the laboratory, results were read after 16–20 h incubation according to the latest version of the RAST breakpoint tables [16].

In the case of polymicrobial BC growth or unreliable RAST results due to high or low bacterial inoculum, an additional standard antibiogram was performed the next day on isolated colonies and interpreted according to the regular EUCAST breakpoints [17]. The minimum inhibitory concentrations (MICs) were determined using the Vitek 2 system (GN cards) or MIC test strips (Liofilchem, Roseto degli Abruzzi, Italy). Colistin MIC was determined by broth microdilution (BMD) Micronaut test (Merlin Diagnostika GmbH, Bornheim, Germany).

AST was supplemented, when necessary, with additional resistance testing as a part of routine laboratory practice [18,19]. Eazyplex Acineto assay (Amplex Diagnostics, Gieβen, Germany) based on the loop-mediated isothermal amplification (LAMP) technique performed on a Genie II device (OptiGene, Gieβen, Germany) was used for detection of OXA variants (OXA51, OXA23, OXA40 and OXA58-like) and NDM in *Acinetobacter.* Colistin resistance gene *mcr-1* was detected by FilmArray BCID2 panel (Biofire, BioMérieux Marcy l’Etoile, France). The genes encoding SHV and SHV-ESBL beta-lactamases were determined by PCR using protocols and conditions previously described [7].

#### 2.3.1. Screening and Confirmation of ESBLs

ESBLs were screened by disc diffusion and confirmed by combination disc test (CDT) (Liofilchem, Roseto degli Abruzzi, Italy) [19]. The inhibition zone around the cefotaxime (30 mg) and ceftazidime (30 mg) disc combined with clavulanic acid is compared with the zone around the cephalosporin disc alone. The test was considered positive if the inhibition zone diameter was ≥5 mm larger with clavulanic acid than without. Eazyplex SuperBug CRE assay (Amplex Diagnostics, Gieβen, Germany) was used for the further identification of ESBL enzyme genes from CTX-M-1 and CTX-M-9 groups.

#### 2.3.2. Screening and Confirmation of Carbapenemase Production

Carbapenemase production was screened using the EUCAST screening cut-off values for carbapenemase-producing *Enterobacterales*, and confirmed by OKNV RESIST-5 assay (CORIS, BioConcept, Gembloux, Belgium) [17]. Carbapenemase gene variants Verona integron-encoded metallo-beta-lactamase (VIM-1-37), New Delhi metallo-beta-lactamase (NDM-1-7), *Klebsiella pneumoniae* carbapenemase (KPC) and OXA-48-like were further analysed by Eazyplex SuperBug CRE assay.

### 2.4. Molecular Mouse System

The Molecular Mouse system (Alifax, Polverara, Italy) is fully automated rapid PCR-based test system for the qualitative detection of DNA targets by ready-to-use lab-on-chip cartridges with lyophilized reagents [13]. As the 64 molecular targets of sepsis panel are grouped in 5 different cartridges (GNB identification, GNB resistance, yeast identification, staphylococcus identification and Gram-positive non-staphylococcus identification), prior microscopic examination of the positive BC is required to select the appropriate cartridge for uploaded to the system. One Molecular Mouse software session allows performing up to 6 simultaneous multiplex reactions [13].

Two different cartridges were evaluated in this study: “MM gram neg ID” cartridge for detection of the most common GNB (15 different targets) and “MM gram neg res” cartridge for selected resistance markers (13 targets). The testing was performed according to the manufacturer’s instructions. Briefly, an aliquot (200 μL) of the positive BC was centrifuged to allow plasma/bacteria separation. Second step of centrifugation was carried out to precipitate bacteria, which were afterwards resuspended in DNAse-free water, mixed with loading buffer and, finally, dispended into cartridge wells.

### 2.5. Data Analyses

Identification of BC isolates by Vitek 2 system was defined as reference method. In case of lacking growth or discordant results of MMS and reference methods, FilmArray BCID PCR panel was performed directly out of positive BC bottles.

The results of the genotypic detection of resistance markers obtained by MMS were compared with the genotypic and phenotypic susceptibility results.

MMS results were considered concordant when they were consistent with the results of the reference methods. Positive MMS results that were negative by culture but confirmed to be positive by FilmArray BCID were considered concordant. Negative MMS results that were found to be positive by culture-based methods because the causative pathogens were not included in the “MM gram neg ID” panel were excluded from the further study. Student’s *t*-test was used to compare the mean values of time to results between MMS and conventional reference methods, with significance set at *p* < 0.05. The standard two-by-two contingency table method with Clopper–Pearson confidence intervals is used to assess the sensitivity and specificity of the assays.

### 2.6. Retrospective Analysis of Potential Clinical Impact

The clinical data of patients with BSI episodes were collected retrospectively using electronic medical records. The complete medical history of the BSI episode was reviewed during a multidisciplinary meeting with members of the AMS team. For each BSI episode, it was determined whether results obtained by the MMS panel would have led to changes in the patient’s treatment: (1) would bacterial identification lead to optimisation of antimicrobial therapy; (2) would the antimicrobial resistance gene detection lead to optimisation of antimicrobial therapy? We have defined antimicrobial optimisation as the improvement of a selected treatment regimen that is both effective and appropriate to treat the individual patient’s infection while minimising the development of drug resistance. This may include escalation or de-escalation of antibiotics depending on the type of pathogen. Escalation corresponded to the administration of an effective therapy if none had been started or to broadening the antibacterial spectrum. De-escalation was defined as a narrowing of the antibacterial spectrum or the discontinuation of at least one antibiotic. No personal data were collected, and the study only involved the reuse of existing data.

## 3. Results

During the study period, 80 positive blood cultures with microscopically detected GNB were included in our analysis. Two samples were found to be negative using the MMS “MM gram neg ID” cartridge as the causative pathogens were not included in the panel (*Pseudomonas putida*, n = 1 and *Acinetobacter calcoaceticus*, n = 1). Therefore, these two BC samples were excluded from the further study, and 78 in total were further analysed.

### 3.1. Identification of Gram-Negative BSI Pathogens

Of these 78 BC bottles included in the study, 74 samples were monomicrobial, and 4 were polymicrobial (two or three GNB isolated per BC sample).

The MMS “MM gram neg ID” cartridge correctly identified the isolates in 77/78 positive BC samples at the genus and species level (Table 1). Only one isolate (*Klebsiella aerogenes*) was identified by the MMS at the family level (*Enterobacteriaceae*). The “MM gram neg ID” panel achieved a sensitivity of 98.7% (93.1–99.9; 95% CI) and a specificity of 100% (95.4–100; 95% CI) for the organisms covered. Average PCR cycle threshold (Ct) values calculated from the provided Ct values of a given species are also shown. Most of the BC samples (85.9%) were aerobic, but MMS also achieved accurate identification from anaerobic bottles.

In the polymicrobial samples (routine culture, n = 4), the results obtained with the MMS method agreed in two, while a discrepancy was found in the other two cases (Table 2). In one BC sample with the discrepancy, the MMS detected *Stenotrophomonas maltophilia*, which was not detected by the routine culture. In the other sample, two GNBs (*K. pneumoniae* and *K. aerogenes*) were detected by MMS that were categorised as one isolate at first using the reference culture method (*Enterobacter cloacae*). For both samples with discrepancies, the MMS results were confirmed as true positive with the FilmArray BCID panel.

### 3.2. Detection of Resistance Genes

Out of 78 positive BC bottles included in the study, 20 BC samples with MDR GNB isolates confirmed by culture-based and molecular-based reference methods were selected for the detection of resistance genes by MMS. A total of 18 BCs with isolated *Enterobacterales* and 2 BCs with *A. baumannii* were analysed for 13 resistance markers using the “MM gram neg res” assay (Table 3). Antimicrobial resistance genes were correctly detected in 19 BCs, i.e., in all *Enterobacterales* (18/18 samples) and in 1 of 2 analysed BCs with *A. baumannii*. Overall susceptibility of assay was 100% (90.9–100; 95% CI), and overall specificity was 100% (90.9–100; 95% CI).

All of the ESBLs associated genes detected were CTX-M-1/9 (n = 17/20), alone or in combination with SHV-type β-lactamase gene or/and with one of the three different types of carbapenemase resistance genes (OXA-48: n = 6; KPC: n = 1; NDM: n = 1). The MMS also accurately detected the *mcr-1* colistin-resistance gene in BC with colistin-resistant *E. coli*. Furthermore, the MMS found the carbapenemase gene OXA-23 in a carbapenem-resistant *A. baumannii*-positive BC sample. In another *A. baumannii* positive BC sample, MMS did not detect any resistance gene.

### 3.3. Performance of MMS Identification and Resistance Gene Detection Assays for Each Target Compared to Reference Methods

The results from each target of MMS-GNB ID and -GNB resistance assays were compared to reference methods (Table 4). The positive BCs did not contain all tested ID/resistance determinants. With regard to monomicrobial BSI, no false negative results were obtained for the 82 GN bacterial strains and 20 resistance genes for which a target was present in the MMS-GNB ID or MMS-GNB resistance panel. One isolate (*K. aerogenes*) was identified with MMS at the family level (*Enterobacteriaceae*). On the other hand, *S. maltophilia* identified with MMS and confirmed with reference molecular assay, was not detected in the polymicrobial sample by routine culture. Neither the MMS-GNB ID nor MMS-GNB resistance panel gave false-positive results. The absence of *Haemophilus influenzae*, *Neisseria meningitidis* and *Salmonella typhi*, as well as organisms harbouring CMY-2 AmpC beta-lactamase and carbapenemases of the VIM and IMP type, made an evaluation of these targets impossible.

### 3.4. Time to Results of MMS Testing and Preliminary Results of Reference Methods

The average time from BC bottle processing after flagging as positive to the MMS results was 1 h (5 min hands-on time + 55 min testing). The average time to MMS results was significantly shorter (*p* < 0.001) than the 16 h required for the preliminary results obtained by reference methods (Table 5).

### 3.5. Potential Impact of MMS Results on Empirical Antimicrobial Therapy

The results of the MMS identification/resistance were analysed, taking into account the initial empirical treatment (Table 6). Moreover, 20 BC samples with detected MDR pathogens as well as six BC samples with detected *P. aeruginosa* and *S. maltophilia* were considered for a possible change in therapy based on the MMS results.

The MMS results could have prompted an early change in empirical therapy in 15/26 (57.7%) patients. In 3 (11.5%) cases, empirical therapy would be de-escalated, while in another 12 (46.1%) cases, therapy would be switched to a broader spectrum or another drug class. In 11 (42.3%) cases, the initial empirical antibiotic therapy was appropriate.

### 3.6. Hospital Wards with Positive BCs

The BC samples were mostly from the emergency department (n = 28) and ICU (n = 22) (Table 7). The most resistant GNB strains (OXA-23, OXA-48, NDM, KPC) (n = 5/20) were detected in BC samples received from the ICU.

## 4. Discussion

Early and accurate diagnosis of BSI is crucial for initiating appropriate antimicrobial treatment. Since current culture-based methods often require 24 to 72 h to obtain results, empirical broad-spectrum antibiotics are usually administered within the first hour of clinical suspicion of sepsis to avoid delays in treatment. Given the rising trend of GNB resistance to beta-lactams, selecting appropriate empirical antibiotic therapy has become even more challenging, leading to the risk of under- or over-treatment.

Croatia is one of the European countries with a high resistance rate in invasive GNB strains [5]. In 2023, the proportion of the most common ESBL-producing *Enterobacterales* (*E. coli* and *K. pneumoniae*) among invasive strains was 27% and 54%, respectively. Additionally, carbapenem-resistant *K. pneumoniae* (mainly OXA-48 carbapenemase producers) have been continuously spreading in Croatia since the COVID-19 pandemic [6]. Among invasive *K. pneumoniae* strains, the percentage of carbapenemase producers was 34% in 2023. The distribution of carbapenemases in invasive *K. pneumoniae* strains in Croatia over a three-year period is shown in Table 8.

Carbapenem resistance in *A. baumannii* has become a growing problem over the last decade and remains extremely high (97%). As reported in a Croatian national survey, carbapenem resistance in A. *baumannii* is primarily associated with the OXA-23 enzyme [21]. Considering the current situation, carbapenem-resistant *K. pneumoniae* and MDR *A. baumannii* represent the greatest challenge for infection control and AMS teams in Croatia. However, carbapenem resistance in *P. aeruginosa* should not be overlooked, as, except for *K. pneumoniae*, the most significant increase in carbapenem resistance in our country in 2023 was recorded in *P. aeruginosa* (30%), with the number of reported isolates being the highest in the last decade.

Although some new antimicrobials (ceftazidime/avibactam, ceftolozane/tazobactam, imipenem/relebactam, meropenem/vaborbactam, aztreonam/avibactam, cefiderocol) covering MDR GNB are available, they should be used in accordance with AMS principles to reduce antibiotic selection pressure. These new antibiotics have varying activity against specific GNB resistance patterns, contributing to the complexity of choosing appropriate empirical therapy. For example, ceftazidime-avibactam is recommended for OXA-48 producers but not for metalo-beta-lactamase (MBL) producers. Cefiderocol is the first choice for NDM and other MBL producers. Aztreonam-avibactam was approved by the European Commission in 2024 primarily for the treatment of infections caused by MBL-producing GNB when other treatment options are limited. These new agents should preferably be administered empirically for a few hours until targeted therapy can be introduced based on microbiology results [22]. This highlights the need for a faster diagnostic approach to BSI.

Rapid identification of pathogens and detection of antibiotic resistance through molecular testing supports effective ASM programmes, enabling early initiation or timely adjustment of antibiotic therapy. Additionally, the negative predictive value of molecular tests is extremely valuable in clinical judgement about the need for antimicrobial therapy. Evaluation of several commercial multiplex PCR-based tests has shown satisfactory clinical performance and good agreement with conventional culture-based methods for identification and antimicrobial susceptibility [10,11,12].

In this study, we evaluated MMS, a new CE-IVD labelled multiplex PCR system for BSI diagnosis. Two types of cartridges were included, one for GNB identification with 15 different targets and the other for 13 antimicrobial resistance gene targets. The MMS correctly identified GNB at the genus and species level in all but one BC sample, in which *K. aerogenes* was identified only at the family level (*Enterobacteriaceae*). Therefore, the MMS assay achieved an overall sensitivity of 98.7% and a specificity of 100% for the identification of GNB contained in the panel. The MMS assay showed a similar high performance to the most established commercial molecular tests for BSI diagnosis [23]. Although *Enterobacterales* outnumbered non-*Enterobacterales* in the analysed positive BC samples, MMS correctly identified several non-fermentors. However, *P. putida* and *A. calcoaceticus* (BC samples excluded from the study) were not detected, as they are not included in the MMS panel. Therefore, in future updates of the MMS panel, it might be beneficial to consider expanding the panel with additional non-fermenters.

According to the European Antimicrobial Resistance Surveillance Network (EARS-Net) 2023 data, the two critical pathogens for BSI with the highest incidence of antimicrobial resistance were *E. coli* and *K. pneumoniae* [24]. Therefore, the high proportion of *E. coli* and *K. pneumoniae* detected in the BC samples in our study is not surprising. Due to its high resistance rate, *K. pneumoniae* has been identified as a pathogen of increasing importance and an independent risk factor for bacteraemia-related deaths [25].

The role of MMS in the detection of all pathogens present in polymicrobial BCs is particularly significant, showing higher sensitivity than the culture-based method. The failure of the cultivation method may be due to the uneven density or different growth rates of individual GNBs. As a result, slower-growing bacteria may not have multiplied sufficiently for successful cultivation when the BC is flagged as positive. In contrast to MMS, some other rapid molecular platforms have shown lower sensitivity in detecting polymicrobial sepsis cases [23].

In view of the continuing increase in carbapenem resistance in GNB, the timely characterisation of resistance mechanisms is crucial for the clinical use of new antimicrobial agents in potentially life-threatening BSI [10]. The MMS comprises 13 different resistance markers: 11 for beta-lactamases genes and 2 for *mcr*-mediated colistin resistance. In addition to the most common beta-lactamase gene SHV and ESBL genes (SHV ESBL, CTX-M-1/9, CTX-M-2/8), MMS can identify the ampC beta-lactamase CMY-2 gene. The MMS panel covers class A and D carbapenemases (KPC, OXA-48 and OXA-23), metalo-beta-lactamases (IMP, VIM, NDM) and colistin resistance genes (*mcr-1*, *mcr-2*).

Selected BC samples containing MDR *Enterobacterales* or CRAB were subjected to MMS detection of resistance genes. Various resistance genes were detected in all selected BCs with MDR *Enterobacterales* (18/18 samples) and in one of two analysed BCs with CRAB. However, not all possible resistance genes included in the MMS panel could be evaluated due to limited representation in the selected BCs. All *K. pneumoniae* strains carried the SHV gene, which is consistent with intrinsic SHV-1 or SHV-11 beta-lactamase production. In addition, the CTX-M gene was detected in all BCs with ESBL-positive *E. coli* or *K. pneumoniae*, which is consistent with previous reports of the prevalence of MDR *K. pneumoniae* with the CTX-M beta-lactamase in Croatian hospitals [26,27].

The MMS detected three different types of carbapenemase genes in *K. pnemoniae*: the oxacillinase OXA-48 gene in six cases, while the KPC and NDM genes were present in one case each. Multidrug resistance in OXA-48-producing *K. pneumoniae* often results from the co-production of ESBLs, which is confirmed in our study, too. OXA-48, combined with CTX-M type ESBL, presented the most important contributor to carbapenem resistance in this strain. The latest report from the Croatian national surveillance system shows that the situation is similar in most Croatian hospitals, long-term care facilities and in the community [6,28].

In a BC with confirmed CRAB, the MMS detected the carbapenemase gene OXA-23. As previously reported, carbapenem resistance in *A. baumannii* in Croatia is mainly associated with the enzyme OXA-23 [29,30]. In another BC sample with detected *A. baumannii*, no resistance gene was detected by MMS. This was not surprising, as many MDR non-lactose fermenters have variable resistance mechanisms and complex genotype-phenotype relationships. Therefore, the MMS assay has achieved a sensitivity of 100% and a specificity of 100% for the detection of all present GNB resistance genes. We note that the absence of organisms displaying CMY-2 AmpC beta-lactamase, SHV ESBL beta-lactamase and VIM and IMP carbapenemases made their evaluation impossible.

Without a doubt, correct and fast detection of genes, at least the most important ones, that encode carbapenemases is certainly a significant feature of MMS.

Although the local resistance rate is not high, the occurrence of *Enterobacterales* resistance to colistin is worrying [31]. Colistin is a last-resort antibiotic for treating MDR GNB infections. Therefore, awareness of the presence of colistin resistance in carbapenem-resistant *Enterobacteriaceae* is of particular concern. The plasmid-linked *mcr* gene for colistin resistance has great potential for rapid spread. The *mcr-1* gene is the most common variant in *Enterobacterales*, particularly in *E. coli* [32]. In our study, the MMS assay detected colistin resistance gene *mcr-1* in an *E. coli* strain. This finding was confirmed by the reference method. Although a low prevalence of *mcr*-mediated colistin resistance has been documented in Europe, the actual incidence may have been underestimated as surveillance data are limited to a small number of studies [24]. We emphasise the lack of survey data on the prevalence of *mcr* genes in *Enterobacterales* strains from our country. Therefore, *mcr*-mediated colistin resistance should be monitored at local and global levels through active, targeted screening.

Conventionally, positive BC is subcultured for 18–24 h to obtain pure colony growth. Then, it takes a further 24 h to obtain AST results. The EUCAST rapid AST method, which is performed directly from positive BCs with an incubation time of 4 to 8 h, is now used in many laboratories. Although a rapid result within the same day is always preferable for the patient, in clinical laboratories with limited opening hours, the extended incubation time of 16–20 h is used to complement the standard RAST. The MMS time to results was compared to the time to preliminary results of the rapid identification/AST methods, which takes an average of 16 h. In this study, the simultaneous performance of MMS for GNB identification and gene resistance testing shortened the time to results by at least 15 h before the results of the culture method. This means that MMS can reduce the time-to-optimised-therapy from 16 h to 1 h.

The ultimate goal of our study was to determine whether the results of rapid identification and detection of resistance genes using MMS could affect the applied empirical antimicrobial therapy. Analysing the patients’ medical history, we found that, using MMS, the rapidly obtained data could have been the reason for changing the initial antimicrobial therapy used in more than half of the cases. In 11.5% of suspected BSI cases, empirical therapy would be de-escalated, while in a further 46.1%, therapy would be switched to a broader spectrum. In slightly less than half of the suspected BSI cases, the initial empirical antibiotic therapy was appropriate. Four BSI cases caused by non-fermenting GNB were found to have inappropriate empirical coverage. Similarly, Sadyrbaeva-Dolgova et al. reported the low accuracy of empirical treatment of BSI due to non-fermenting GNB in their retrospective, observational cohort study [33].

In our recently published, retrospective cross-sectional study carried out in septic ICU patients, we found that around one-third (32%) of patients with confirmed GNB BSI received inappropriate antimicrobial therapy [8]. The higher percentage of cases with changed initial empirical therapy observed in this study (57.7%) should be interpreted with caution, considering that, for the purposes of MMS validation, only BCs with MDR GNB were selected here. Moreover, the BCs analysed in the present study were collected from different hospital wards, not only from the ICU, where patients are at higher risk of MDR bacterial infections, and broad-spectrum antibiotics or combined antibiotic regimens are frequently used as empirical therapy.

Rapid microbiological technologies can contribute to the quick identification of causative agents and major genetic determinants of antimicrobial resistance, enabling the early selection of appropriate antimicrobial therapy and, at the same time, reducing antibiotic exposure. The use of MMS assays to identify pathogens from positive BCs shortens both turnaround and hands-on time. The advantages of different multiplex PCR assays over conventional methods are speed and sensitivity, but the high cost limits their use in low- and middle-income countries. However, MMS offers the possibility to select the appropriate cartridge based on Gram-stain, which helps reduce the cost of the analysis. Our results confirm the good performance of MMS in the identification of GNB and the detection of antimicrobial resistance. The evaluated assays showed considerable potential as a rapid tool in the diagnosis of GNB BSI. To our knowledge, only one clinical study has validated MMS assays to date. Our results are consistent with the study by Mauri et al., who found a high concordance between MMS performance and conventional routine microbiological methods [34].

However, it should be noted that they evaluated the analytical performance of MMS in BC samples from hospitals in northern Italy. The estimated incidences of BSI with resistant bacteria varied widely between European countries [5,24]. For this reason, the pathogens identified and the resistance genes detected in these two studies differed. Another difference between our and the Italian study is the research setting. While Mauri et al. analysed BC samples from critically ill patients, we investigated BC samples from patients treated in different hospital wards. In our study, most of the BC samples came from emergency departments and ICUs, and the rest from general wards. Therefore, the GNB we identified actually represents the epidemiology of GNB in the entire hospital, not just in the ICU. Like in our study, Mauri et al. also found a high proportion of CTX-M-1/9 ESBL genes, which is not surprising given the high prevalence of these genes in *K. pneumoniae* species in Italy and Croatia [24]. However, probably due to the different epidemiology, they found only one oxacillinase OXA-48 gene among the *K. pneumoniae* isolates, while no other carbapenemases (NDM, KPC, OXA-28) or *mcr*-1 genes were detected in their evaluation [34].

The present study had several limitations. MMS has the ability to identify only on-panel organisms and relies on Gram-staining. However, choosing an appropriate assay based on microscopy results helps reduce testing costs, potentially increasing the applicability of the assays. Another limitation was the lack of samples positive for some of the pathogens and resistance genes included in the MMS assays, making it impossible to draw conclusions about the assay’s performance in detecting them. Future studies with larger sample sizes are needed for clinical evaluations of these specific targets. Additionally, although the MMS assay correctly detected all present resistance genes, it can only identify a limited number of resistance genes and does not account for all resistance mechanisms (e.g., loss of porins and overexpression of efflux pumps). Finally, retrospective analysis of the potential clinical effects of MMS may be subject to uncontrolled confounding and bias, so further prospective studies are necessary to clarify this point.

## 5. Conclusions

Despite these limitations and in light of our study objectives, MMS showed good results in the rapid detection of Gram-negative pathogens and their resistance genes directly from positive BC. The fast results obtained with MMS may have led to changes in empirical therapy for a significant proportion of BSI episodes caused by MDR pathogens. The addition of molecular testing as a complementary tool to culture-based diagnosis of BSI may shorten turnaround time, reduce overuse of antibiotics and improve patient outcomes.

## Figures and Tables

**Table 1:** Identification of Gram-negative BSI pathogens in aerobic and anaerobic blood cultures.

Molecular Mouse Result (n = 82)	Type of Blood Culture Bottles (n = 78) AER = 67, ANA = 11	Ct Value (Mean ± SD)
*Enterobacterales* (n = 73)		
*E. coli/Shigella* spp. (n = 29)	AER = 24; ANA = 5	26.3 ± 2.3
*K. pneumoniae* (n = 22)	AER = 19; ANA = 3	26.9 ± 2.8
*Proteus mirabilis* (n = 8)	AER = 6; ANA = 2	24.5 ± 1.2
*Enterobacter cloacae* (n = 7)	AER = 6; ANA = 1	26.4 ± 3.7
*Serratia marcescens* (n = 3)	AER = 3; ANA = 0	26.5 ± 1.3
*K. oxytoca* (n = 2)	AER = 2; ANA = 0	29.5 ± 0.0
*^a^ Enterobacteriacae* (n = 1)	AER = 1; ANA = 0	27.5 ± 2.4
*K. aerogenes* (n = 1)	AER = 1; ANA = 0	28.1
Non-*Enterobacterales* (n = 9)		
*P. aeruginosa* (n = 4)	AER = 4; ANA = 0	27.1 ± 2.4
*^b^ S. maltophilia* (n = 2)	AER = 2; ANA = 0	30.4 ± 4.4
*A. baumannii* (n = 3)	AER = 3; ANA = 0	30.9 ± 1.0

Notes: *^a^* one isolate (*K. aerogenes*) was identified with MMS at the family level, *^b^ S. maltophilia* identified with MMS in the polimicrobial sample was not detected by the routine culture. Abbreviations: AER, aerobic; ANA, anaerobic.

**Table 2:** Identification of polymicrobial BSI pathogens.

Molecular Mouse System	Routine Culture Method
*E. coli/Shigella* spp., *A. baumannii*	*E. coli*, *A. baumannii*
*K. oxytoca*, *P. mirabilis*, *S. maltophilia*	*K. oxytoca*, *P. mirabilis*
*K. pneumoniae* , *K. aerogenes*	*E. cloacae*
*K. pneumoniae*, *E. coli*	*K. pneumoniae*, *E. coli*

Notes: Bacteria revealed only by the Molecular Mouse system are underlined.

**Table 3:** Resistance genes detected by the Molecular Mouse system.

Molecular Mouse Result (n = 20)	Routine Culture Method
*E. coli*/*Shigella* spp. SHV, CTX-M-1/9 groups (n = 1)	ESBL-producing *E. coli*
*E. coli*/*Shigella* spp. CTX-M-1/9 groups (n = 3)	ESBL-producing *E. coli*
*K. pneumoniae* SHV, CTX-M-1/9 groups (n = 3)	ESBL-producing *K. pneumoniae*
*K. pneumoniae* SHV, CTX-M-1/9 groups, OXA-48 (n = 6)	Carbapenem-resistant *K. pneumoniae* (OXA-48 enzyme)
*K. pneumoniae* SHV, CTX-M-1/9 groups, NDM (n = 1)	Carbapenem-resistant *K. pneumoniae* (NDM enzyme)
*K. pneumoniae* SHV, CTX-M-1/9 groups, KPC (n = 1)	Carbapenem-resistant *K. pneumoniae* (KPC enzyme)
*A. baumannii* OXA-23 (n = 1)	Carbapenem-resistant *A. baumannii* (OXA-23 enzyme)
*^a^ A. baumannii* (n = 1)	Carbapenem-resistant *A. baumannii* (unknown mechanism)
*E. coli mcr-1* (n = 1)	Colistin-resistant *E. coli*
*K. oxytoca* CTX-M-1/9 groups (n = 1)	ESBL-producing *K. oxytoca*
*P. mirabilis* CTX-M-1/9 groups, (n = 1)	ESBL-producing *P. mirabilis*

Notes: *^a^* resistance gene was not detected. Abbreviations: CTX-M, cefotaxime-Munich; ESBL, Extended-spectrum beta-lactamase; KPC, *Klebsiella pneumoniae* carbapenemase; *mcr*, mobilised colistin resistance; NDM, New Delhi metallo-beta-lactamase; OXA, oxacillinase; SHV, sulfhydryl reagent variable.

**Table 4:** Performance of MMS identification and resistance gene detection assays for each target in comparison to reference methods.

Molecular Mouse Targets (n = 82)	Reference Methods	Sensitivity (%) [95% CI]	Specificity (%) [95% CI]
GNB identification			
*E. coli/Shigella* spp.	*E. coli*	29/29 (100) [88.1–100]	53/53 (100) [93.3–100]
*K. pneumoniae*	*K. pneumoniae*	22/22 (100) [84.6–100]	60/60 (100) [94.0–100]
*P. mirabilis*	*P. mirabilis*	8/8 (100) [63.1–100]	74/74 (100) [95.1–100]
*Proteus* spp.	No other species	8/8 (100) [63.1–100]	74/74 (100) [95.1–100]
*E. cloacae*	*E. cloacae*	7/7 (100) [59.0–100]	75/75 (100) [95.2–100]
*S. marcescens*	*S. marcescens*	3/3 (100) [29.2–100]	79/79 (100) [95.4–100]
*K. oxytoca*	*K. oxytoca*	2/2 (100) [15.8–100]	80/80 (100) [95.5–100]
*K. aerogenes*	*^a^ K. aerogenes*	1/2 (50) [1.3–69.7]	80/80 (100) [95.5–100]
*P. aeruginosa*	*P. aeruginosa*	4/4 (100) [39.8–100]	78/78 (100) [95.4–100]
*S. maltophilia*	*^b^ S. maltophilia*	2/2 (100) [15.8–100]	80/80 (100) [95.5–100]
*A. baumannii*	*A. baumannii*	3/3 (100) [25.2–100]	79/79 (100) [95.4–100]
*Haemophilus influenzae*	None		82/82 (100) [95.6–100]
*Neisseria meningitidis*	None		82/82 (100) [95.6–100]
*Salmonella typhi*	None		82/82 (100) [95.6–100]
GNB resistance			
SHV	*K. pneumoniae (11), E. coli (1)*	12/12 (100) [73.5–100]	8/8 (100) [63.1–100]
CTX-M-1/9	*K. pneumoniae (11), E. coli (4),*	17/17 (100) [80.5–100]	3/3 (100) [29.2–100]
	*K. oxytoca (1), P. mirabilis (1)*		
OXA-48	*K. pneumoniae (6)*	6/6 (100) [54.1–100]	14/14 (100) [76.8–100]
NDM	*K. pneumoniae (1)*	1/1 (100) [2.5–100]	19/19 (100) [82.4–100]
KPC	*K. pneumoniae (1)*	1/1 (100) [2.5–100]	19/19 (100) [82.4–100]
OXA-23	*A. baumannii (1)*	1/1 (100) [2.5–100]	19/19 (100) [82.4–100]
*mcr-1*	*E. coli (1)*	1/1 (100) [2.5–100]	19/19 (100) [82.4–100]
*mcr-2*, VIM, IMP, SHV-ESBL,	None		20/20 (100) [83.2–100]
CTX-M-2/8, CMY-2	None		

*^a^* one isolate (*K. aerogenes*) was identified with MMS at the family level (*Enterobacteriaceae*), *^b^ S. maltophilia* identified with MMS in the polymicrobial sample was not detected by the routine culture, but the finding was confirmed with FilmArray blood culture identification (BCID) panel. Abbreviations: GNB, Gram-negative bacteria.

**Table 5:** Time to results of MMS testing and preliminary results of reference method.

	Conventional Method (n = 78)	MMS (n = 78)	*p*
Time to results, mean (SD), hours	16.0 (4.6)	1 (0.0)	<0.001

**Table 6:** Potential impact of the MMS results on the initial empirical therapy.

Molecular Mouse Result	Appropriate Therapy—No Change Required (n = 11/26)	Potential Therapy Change (n = 15/26)		
		Therapy De-Escalation	Need to Expand the Antimicrobial Coverage	Therapy Change- Total
*Enterobacterales* total (n = 18)				
*Enterobacterales* ESBL/non-carbapenemase (n = 10)	6	2	2	4
*Enterobacterales* ESBL + carbapenemase (n = 8)	2	1	5	6
Non-*Enterobacterales* (n = 8)				
*A. baumannii* OXA-23 (n = 1)	0	0	1	1
*A. baumannii* (n = 1)	1	0	0	0
*P. aeruginosa* (n = 4)	2	0	2	2
*S. maltophilia* (n = 2)	0	0	2	2
Total (n = 26)	11	3	12	15

Abbreviations: ESBL, extended-spectrum beta-lactamase; OXA, oxacillinase.

**Table 7:** Summary of isolates and resistance genes identified from blood cultures from different hospital wards.

Number of Positive BC Samples from Clinical Wards (n = 78)	Species of Gram-Negative Bacteria	Resistance Genes
Emergency (n = 28)	*E. coli*, *K. pneumoniae*, *K. oxytoca*, *E. cloacae*, *K. aerogenes*, *S. maltophilia*, *P. mirabilis*, *P. aeruginosa*	SHV, CTX-M 1/9
ICU (n = 22)	*E. coli*, *K. pneumoniae*, *E. cloacae*, *P. aeruginosa*, *A. baumannii*	SHV, CTX-M 1/9, OXA-23, *mcr*-1, OXA-48, NDM, KPC
Nephrology (n = 5)	*P. aeruginosa*, *S. marcescens*, *P. mirabilis*, *K. pneumoniae*	
Cardiology (n = 4)	*E. coli*, *S. marcescens*	
Pediatry (n = 4)	*E. coli*, *P. aeruginosa*, *K. pneumoniae*, *E. cloacae*	
Urology (n = 3)	*E. coli*, *K. pneumoniae*	SHV, CTX-M 1/9, OXA-48
Gastroenterology (n = 3)	*E. coli*, *K. pneumoniae*	
Infectology (n = 3)	*E. coli*	
Cardiothoracic surgery (n = 3)	*K. pneumoniae*, *E. cloacae*	SHV, CTX-M 1/9, OXA-48
Neurology (n = 2)	*K. pneumoniae*, *P. mirabilis*	SHV, CTX-M 1/9, OXA-48
Haematology (n = 1)	*E. coli*	

Abbreviations: BC, blood culture; ICU, intensive care unit; CTX-M, cefotaxime-Munich; KPC, *Klebsiella pneumoniae* carbapenemase; *mcr*, mobilised colistin resistance; NDM, New Delhi metallo-beta-lactamase; OXA, oxacillinase; SHV, sulfhydryl reagent variable.

**Table 8:** Distribution of carbapenemases in *K. pneumoniae* isolated from BSIs in Croatia from 2021 to 2023 [6,20,21].

Carbapenemase	2021 n (%)	2022 n (%)	2023 n (%)
OXA-48	3092 (87.8%)	3233 (91.2%)	3248 (68.1%)
KPC	368 (10.5%)	200 (5.6%)	117 (2.4%)
MBL			
NDM	46 (1.3%)	84 (2.4%)	118 (2.5%)
VIM	14 (0.3%)	28 (0.7%)	1287 (26.9%)

Abbreviations: BSI, bloodstream infection; MBL, metalo-beta-lactamases; KPC, *Klebsiella pneumoniae* carbapenemase; NDM, New Delhi metallo-beta-lactamase; OXA, oxacillinase; VIM, Verona integron encoded metallo-beta-lactamase.

## Data Availability

Data supporting this article is available from the corresponding author upon reasonable request.

## Abbreviations

The following abbreviations are used in this manuscript:

AMS	Antimicrobial stewardship
AST	Antimicrobial susceptibility testing
BC	Blood culture
BSI	Bloodstream infections
CRAB	Carbapenem resistant *Acinetobacter baumannii*
ESBL	Extended-spectrum beta-lactamases
EUCAST	European Committee on Antimicrobial Susceptibility Testing
GNB	Gram-negative bacteria
ICU	Intensive Care Unit
MDR	Multidrug-resistant
MMS	Molecular Mouse System
PCR	Polymerase chain reaction
WHO	World Health Organization
